# Effect of PAK Inhibition on Cell Mechanics Depends on Rac1

**DOI:** 10.3389/fcell.2020.00013

**Published:** 2020-01-28

**Authors:** Claudia Tanja Mierke, Stefanie Puder, Christian Aermes, Tony Fischer, Tom Kunschmann

**Affiliations:** Faculty of Physics and Earth Science, Peter Debye Institute of Soft Matter Physics, Biological Physics Division, University of Leipzig, Leipzig, Germany

**Keywords:** cell migration and invasion, cell deformation (compliance), Rac1, PAK1–3, optical cell stretching, magnetic tweezer, 3D collagen matrices, PAK inhibitors

## Abstract

Besides biochemical and molecular regulation, the migration and invasion of cells is controlled by the environmental mechanics and cellular mechanics. Hence, the mechanical phenotype of cells, such as fibroblasts, seems to be crucial for the migratory capacity in confined 3D extracellular matrices. Recently, we have shown that the migratory and invasive capacity of mouse embryonic fibroblasts depends on the expression of the Rho-GTPase Rac1, similarly it has been demonstrated that the Rho-GTPase Cdc42 affects cell motility. The p21-activated kinase (PAK) is an effector down-stream target of both Rho-GTPases Rac1 and Cdc42, and it can activate via the LIM kinase-1 its down-stream target cofilin and subsequently support the cell migration and invasion through the polymerization of actin filaments. Since Rac1 deficient cells become mechanically softer than controls, we investigated the effect of group I PAKs and PAK1 inhibition on cell mechanics in the presence and absence of Rac1. Therefore, we determined whether mouse embryonic fibroblasts, in which Rac1 was knocked-out, and control cells, displayed cell mechanical alterations after treatment with group I PAKs or PAK1 inhibitors using a magnetic tweezer (adhesive cell state) and an optical cell stretcher (non-adhesive cell state). In fact, we found that group I PAKs and Pak1 inhibition decreased the stiffness and the Young’s modulus of fibroblasts in the presence of Rac1 independent of their adhesive state. However, in the absence of Rac1 the effect was abolished in the adhesive cell state for both inhibitors and in their non-adhesive state, the effect was abolished for the FRAX597 inhibitor, but not for the IPA3 inhibitor. The migration and invasion were additionally reduced by both PAK inhibitors in the presence of Rac1. In the absence of Rac1, only FRAX597 inhibitor reduced their invasiveness, whereas IPA3 had no effect. These findings indicate that group I PAKs and PAK1 inhibition is solely possible in the presence of Rac1 highlighting Rac1/PAK I (PAK1, 2, and 3) as major players in cell mechanics.

## Key Findings (Impact On Science)

• Rac1 and PAK1 act as major players in cell mechanics.
• When cells are in their adhesive state both the group I PAKs and the PAK1 inhibitors function only in the presence of Rac1.

• The Young’s modulus (or stiffness) of adhesive Rac1^–/–^ cells is not altered by both inhibitors of group I PAKs and PAK1, whereas the stiffness of adhesive Rac1^fl/fl^ cells is pronouncedly decreased.
• When the cells are in their non-adhesive state, only the PAK1 inhibitor IPA3, which, in contrast to FRAX597, interferes with inactive PAK1, has an effect on Rac1^–/–^ cells.
• FRAX597 inhibition of the kinase domain of PAK1 reduces the Young’s modulus (stiffness) of Rac1^fl/fl^ cells, but not of Rac1^–/–^ cells independent of their adhesive state.
• The competitive group I PAKs inhibitor FRAX597 reduces invasiveness, whereas the allosteric PAK1 inhibitor IPA3 reduces solely the invasiveness in the presence of Rac1.

## Introduction

The migration and invasion of cells, such as fibroblasts, is facilitated by the polymerization of actin that is regulated by the Rho-family GTPases Rac1 and Cdc42. Both, Rac1 and Cdc42 can evoke the restructuring of the cytoskeletal organization in diverse manners (Kaibuchi et al., 1999; Jaffe and Hall, 2005). These two Rho GTPases act differently in the activation of actin polymerization (Worthylake and Burridge, 2001). The effects of these two Rho GTPases have been investigated primarily on actin filaments, where Rac1 promotes the formation of lamellipodia and Cdc42 supports the formation of filopodia and subsequently, both trigger the protrusive cell activities (Hall, 2005; Hall, 2012). Actin filaments, which assemble lamellipodial mesh structures, are commonly generated through the nucleation of new filaments or branching of older filaments, which is driven by the actin-related protein 2/3 (Arp2/3) complex (Steffen et al., 2006; Nicholson-Dykstra and Higgs, 2008; Suraneni et al., 2012; Wu et al., 2012). Rac1 and Cdc42 cause the activation of the Arp2/3 complex that initiates the nucleation of new actin filaments to create branched actin filament networks (Borisy and Svitkina, 2000; Steffen et al., 2004, 2006). Besides Rac1’s function in providing a branched actin network, Rac1 can uncap barbed ends of pre-existing actin filaments to promote their further growth (Hartwig et al., 1995).

The Rho-GTPases Rac1 and Cdc42 have down-stream effector p21-activated kinases of group I PAKs containing PAK1, 2, and 3. Hence, PAK has been initially reported to function as an interaction molecule for the Rho GTPases Rac1 and Cdc42 (Manser et al., 1994). In detail, the most prominent PAK is PAK1 that contains multiple domains and is made up of 545 amino acids. Moreover, it is composed of an N-terminal regulatory region and a C-terminal catalytic kinase domain (Lei et al., 2000; Zhao and Manser, 2012). The regulatory domain contains, the PBD (synonymously termed CRIB) domain and the auto-inhibitory domain (AID). Initially, in mammals, PAK1 is a founding member of the PAK Ser/Thr protein kinase family that is divided into two subgroups termed group I PAKs (PAK1-3) and group II PAKs (PAK4-6) (Hofmann et al., 2004; Licciulli et al., 2013). The members of the PAK I share 93–95% sequence identity in their kinase domain and hence they are similarly regulated by Rac/Cdc42-GTP binding within the same region in group I PAKs (Jaffer and Chernoff, 2002).

In contrast to the constitutively activated group II PAKs, group I PAKs possess an AID domain (synonymously termed switch domain) and they are activated in their kinase domain (synonymously termed catalytic domain) through Rho GTPases, such as Rac1 and Cdc42 (Kim et al., 2016). The activity of group I PAKs is regulated through a reciprocal auto inhibitory mechanism, whereby two PAK molecules dimerize and become both an inactive kinase domain. In specific detail, the PBD domain overlaps with the AID domain and binds to the kinase domain of the other PAK molecule, which inactivates both dimerized PAK molecules. An individual PAK1 molecule is turned toward an active state by the interaction of its PBD domain and the concomitant interaction with proximal amino acids and phosphoinositides with Cdc42∙GTP and Rac1∙GTP, which induces alterations in the conformation of the catalytic domain (Morreale et al., 2000; Lei et al., 2000). Hence, the AID domain dissociates from the kinase domain that causes further conformational changes in the dimerized PAKs and induces a phosphorylation of both PAK molecules triggering the restoration of their kinase activities. Consequently, the PAK molecules are converted from a dimeric form to a monomeric form (Kumar et al., 2017). Hence, Rac1 and Cdc42 can activate through PAK1 the LIM kinase, which leads to the reduction of cofilin activity through its phosphorylation, and subsequently to increased motility (del Pozo et al., 2000; Pollard and Borisy, 2003). In fact, PAK1 is crucially employed in the regulation of cell motility, signal transduction regulating cytoskeletal dynamical remodeling, the morphology and adhesive state of cells (Sells et al., 1997; Delorme-Walker et al., 2011; Radu et al., 2014). Moreover, PAK1 plays a major role in diseases, such as nervous system disorders including Alzheimer (Ma et al., 2012) and cancer development including malignant progression (Holm et al., 2006; Kamai et al., 2010; Ong et al., 2011; Ye and Field, 2012; Radu et al., 2014).

Group I PAKs can be targeted by multiple inhibitors (Semenova and Chernoff, 2017). These are two different types of PAK inhibitors, such as ATP-competitive (non-covalent) and non-ATP-competitive (allosteric) PAK1 inhibitors (Semenova and Chernoff, 2017). Among the ATP-competitive (non-covalent) ones are the FRAX Aminopyrimidine-based series that are PAK-inhibiting compounds based on a pyrido[2,3- d]pyrimidine-7-one core, such as FRAX597 which potently inhibits PAK1 (IC50 = 7.7 nM), while it displays moderate selectivity against other kinases, such as receptor tyrosine kinases (Licciulli et al., 2013). Among the non-ATP competitive group I PAKs inhibitors are allosteric inhibitors that interact with PAK1 outside of its ATP-binding region. These inhibitors possess a greater selectivity across the kinome compared to ATP-competitive inhibitors, since they interact with less conserved regions of group I PAKs. However, this kind of inhibitors are less potent than ATP-competitive inhibitors, since their targeted protein binding pockets are not so deep and contain not multiple binding sites (Semenova and Chernoff, 2017). The sulfhydryl-containing compound IPA3 (an allosteric inhibitor p21-activated kinase-3) binds covalent to the N-terminal regulatory domain of group I PAKs (Deacon et al., 2008), which prevents the GTPase binding and subsequently the conversion toward a catalytically active state (Viaud and Peterson, 2009). A pronounced inhibition of kinase activity in the presence 10 μM IPA3 has been detected in all three group I PAKs, with the strongest inhibition observed for PAK1 (Deacon et al., 2008). Overall IPA3 represents a distinctive compound that possesses a unique PAK1 binding mode (Semenova and Chernoff, 2017).

However, to our knowledge, the present study is the first to examine the effect of group I PAKs inhibition on cell mechanics in the response to presence or absence of Rac1. Through the use of mouse embryonic fibroblasts, in which Rac1 was knocked out and healthy control cells expressing Rac1, we analyzed the effect of group I PAKs inhibition in dependence of Rac1 on cell stiffness of adhesive cells using magnetic tweezers and non-adhesive cells using optical cell stretching. In fact, we found that in adhesive Rac1^fl/fl^ cells the group I PAKs and PAK1 inhibition reduces cell stiffness, whereas there is no effect on cell stiffness in adhesive Rac1^–/–^ cells. However, the stiffness of non-adhesive cells treated with PAK inhibitors was decreased in Rac1^fl/fl^ cells, whereas the stiffness of non-adhesive Rac1^–/–^ cells was not altered by the competitive FRAX597 inhibitor and rather slightly reduced by the allosteric IPA3 inhibitor. In addition, in both cell types the invasiveness and invasion depths were reduced, after treatment with the FRAX597. In contrast IPA3 treatment had solely an effect of the invasiveness of Rac1^fl/fl^ cells, whereas the invasiveness of Rac1^–/–^ cells was not altered. These results suggest that group I PAKs and PAK1 inhibition by FRAX597 alters cell stiffness (or the Young’s modulus), when Rac1 is expressed, whereas it had no effect on cells where Rac1 is knocked out independent of the adhesive state. In contrast, PAK1 inhibition by IPA3 alters cell stiffness of both cell types solely in their non-adhesive state, whereas only in their adhesive state, the cell stiffness of Rac1 expressing cells was decreased. Elucidating the effect of group I PAKs and PAK1 in dependence of Rac1 on cell mechanics of adhesive and non-adhesive cells and invasion can help to provide more insights of how the mechanics of fibroblasts are regulated.

## Materials and Methods

### Cells and Cell Culture

Mouse embryonic Rac1 wild type (Rac1^fl/fl^ cells) and Rac1 knock-out (Rac1^–/–^ cells) fibroblasts were kindly provided by Prof. Dr. Klemens Rottner and Dr. Anika Steffen and generated as described (Steffen et al., 2013). Fibroblast cells were cultured in Dulbecco’s modified Eagle’s medium (DMEM) containing 4.5 g/L glucose 10% FCS (low endotoxin, <0.1 EU/ml, Biochrom, Berlin, Germany), 2 mM L-glutamine, 0.1 mM MEM non-essential amino acids, 1 mM sodium pyruvate and 1% 100 U/ml penicillin-streptomycin (Gibco, Germany) (Kunschmann et al., 2019). Fibroblasts were analyzed within passages 6 to 30, when they reached 80% confluency. They were harvested with a 0.125%/0.025% Trypsin/EDTA PBS-buffered solution (Biochrom, Berlin, Germany). Other chemical drugs were all obtained from Sigma (Taufkirchen, Germany) unless otherwise stated.

### Analysis of 3D Motility Within Extracellular Matrix Scaffolds

3D extracellular matrices were employed to determine the effect of two PAK inhibitors, FRAX597 and IPA3, on the invasive behavior of Rac1^fl/fl^ and Rac1^–/–^ cells. Since PAK3 is preferable in brain tissue (Molli et al., 2009) and these inhibitors in principle interfere solely with PAK1 and PAK2. However, in mouse embryonic fibroblasts PAK1 is the most abundant form (Nola et al., 2008). For the 3D extracellular matrix, a mixture of type I collagen of rat tail (one third; 4 g/l rat collagen type I, Serva, Heidelberg, Germany) and bovine skin (two thirds; 4 g/l bovine collagen type I, Biochrom, Berlin, Germany) was used.

Each six well plate was filled with the 1.2 ml of the collagen mixture, dH_2_O and 1M phosphate buffer. The final collagen type I gel concentration was 1.5 g/L. The ice-cold collagen gels were polymerized at a pH of 7.4 and an ionic strength of 0.7 at 37°C, 95% humidity and 5% CO_2_ for at least 2 h. Polymerized scaffolds were rinsed several times with PBS and stored overnight in culture medium (Kunschmann et al., 2017, 2019; Fischer et al., 2017). 50.000 cells were placed on top of each matrix scaffold and were incubated for 12 h to allow the cells to adhere on top the matrix. Afterwards the cells were incubated with 1.2 μM FRAX697 or 12 μM IPA3 and for control, cells were incubated with solvent of control vehicle. We have analyzed a wide range of concentrations, such as 1–20 μM for IPA3 (2,2′-dihydroy-1,10-dinaphthyldisulfide) and 0.1–2.0 μM for FRAX597. The concentrations, that were most effective, but displayed less side effects, have been chosen for all experiments. It has been reported that the IPA3 inhibitor has a lower potency compared to ATP-competitive inhibitors, such as FRAX597 (Kim et al., 2016; Semenova and Chernoff, 2017). Other studies used also a 10-fold difference between these two inhibitors (Wang et al., 2016). Cells were cultured 72 h, fixated with 2.5% glutaraldehyde solution (Serva, Heidelberg, Germany) and stained with 4 μg/ml of the Hoechst 33342 dye (Serva, Heidelberg, Germany). The position of invasive cells can be clearly distinguished from that of non-invasive cells, since their nuclei are located below the cell layer of non-invasive cells in the 3D framework of the extracellular matrix. The percentage of invasive and non-invasive cells and their invasion depths were determined in 100 random fields of view. In the central region of each well, 100 image stacks were recorded in a 10 × 10 matrix at 4 μm steps with a 20× objective (DMI8000B, Leica, Wetzlar, Germany) and a 0.55× c-mount adaptor (Leica) for a CCD camera (Orca-R2, Hamamatsu-Photonics, Munich, Germany). The experiments have been repeated three times independently and samples were measured in triplicate. Between 2000 and 17000 cells were analyzed for each condition.

### Magnetic Tweezer Measurements of Adhesive Cells

For magnetic tweezer analysis, 4.5 μm superparamagnetic beads (Dynabeads M450, Sigma Aldrich) were coated with 50 μg/ml human fibronectin (Sigma Aldrich). In detail, firstly, the beads were washed in PBS before the addition of fibronectin. In the next step, the beads were centrifuged at 37°C and incubated at 700 RPM overnight. After centrifugation, the beads were washed twice in PBS and stored at 8°C until usage. Before each measurement, clusters of beads were broken down by rigorously agitating the beads using a vortex mixer.

Secondly, cells were seeded into 35 mm culture dishes for approximately 24 h before the measurement start at a density of about 10^5^ cells per dish. Cells were then incubated at 37°C, 95 humidity and 5% CO_2_. For PAK inhibition, the cell types were incubated with 10 μM IPA3 or 1.2 μM FRAX597 for 2 h at 37°C and 5% CO_2_. Afterwards, coated beads (about 3⋅10^4^ beads per dish) were added to the cells 20 min before a measurement was conducted. The cells were measured for approximately 40 min. The magnetic tweezer was surrounded by an incubation chamber, which was heated to 37°C and filled with 5% CO_2_ enriched air for the measurements. The mechanical properties of the cells were investigated by probing the cells with a constant force of one nanonewton for 2 s. The creep response was fitted with a weak power law:

J(t)=J0(tt0)β

The stiffness was derived as the inverse of the prefactor J_0_. The cell stiffness derived from magnetic tweezer measurements is a shear modulus G evaluated at *t* = 1 s. Assuming a Poisson ratio ν of 0.5 for the cell (Guz et al., 2014; Nijenhuis et al., 2014), the Young’s modulus E can then be estimated by E = 2G(1 + ν). The power law exponent β is a measure for the viscoelastic state of the cells. The creep response of cells with β = 1 indicates that the cells behave completely viscous, while the creep response of cells with β = 0 indicates a purely elastic behavior. Due to the underlying log-normal distribution of the stiffness values, the average elastic modulus of the cell was calculated as the geometric mean. Since the power law exponent β exhibited a normal distribution, the average power law exponent β was calculated as the arithmetic mean. The experiments have been repeated three times independently and samples were measured in triplicate. In specific detail, *n* = 97 Rac1^fl/fl^ control cells, *n* = 107 Rac1^fl/fl^ IPA3 treated cells, *n* = 125 Rac1^fl/fl^ FRAX597 treated cells, *n* = 94 Rac1^–/–^ control cells, *n* = 98 Rac1^–/–^ IPA3 treated cells and *n* = 94 Rac1^–/–^ FRAX597 treated cells were analyzed.

### Immunofluorescence Analysis on 2D Substrates With Confocal Laser Scanning Microscopy

We coated the cleaned glass cover slides with 10 μg/ml laminin for 2 h at 37°C, 95% humidity and 5% CO_2_. They were washed twice with PBS buffer to remove unbounded proteins. 4000 to 8000 cells were pipetted on top of these coated slides and incubated for 16 h under the same conditions. For 2 h, the adherent cells were treated with 1.2 μM FRAX697 or 12 μM IPA3 or solvent of the control vehicle. After slightly washing the glass slides with PBS buffer, the remaining adherent cells were fixated with 4% paraformaldehyde for 10 min at room temperature. Subsequently, cells were washed twice with PBS buffer and blocked with 1% BSA (bovine serum albumin) in PBS buffer for 20 min to reduce background noise of fluorescence dyes. In detail, cells were incubated with 5 units/ml Alexa Fluor 546 Phalloidin (Thermo Fisher Scientific, Waltham, MA, United States) in 1% BSA buffer, 0.25 mg/ml DID (Thermo Fisher Scientific, Waltham, MA, United States) and 0.02 mg/ml Hoechst 33342 (Serva, Heidelberg, Germany) overnight at 4°C to stain their actin filaments and nuclei, respectively. In order to reduce photo bleaching, prolong diamond antifade (Thermo Fisher Scientific, Waltham, MA, United States) was employed and glass cover slides were placed onto a glass slide. These slides were incubated at 4°C for 24 h until a gel-like consistence of prolong diamond antifade was achieved. All slides were sealed with nail polish to analyze them with a confocal laser scanning microscope (TCS SP8, Leica, Wetzlar, Germany). The experiments have been repeated three times independently and 15–20 cells were imaged for each conditions and staining.

### Optical Cell Stretcher Measurements of Non-adhesive Cells

For these cell stretching measurements, cells were cultured 1 day before measurement start to 70% confluency in a T25 cell-culture flask. Cells were harvested with a Trypsin/EDTA (0.125%/0.025%) solution for 4 min and centrifuged at 125g for 5 min. After removing the culture medium, the resulting cell pellet was resuspended in new complete culture medium. Cellular deformation was measured using an automated optical cell stretcher. The optical cell stretcher is a dual-beam laser trap that can trap and deform single suspended cells by optically induced stress. In detail, a microfluidic flow chamber is mounted on an inverted phase-contrast microscope (Zeiss Axio Observer Z1, Zeiss, Oberkochen, Germany) and connected to two laser beams facing each other (Kunschmann et al., 2017, 2019; Mierke, 2019). Individual spherical cells were transported with a microfluidic pump system in front of the two laser beams and cellular deformation was recorded by a CCD camera (Basler A622f, Basler Vision Technologies, Switzerland; Guck et al., 2005; Lincoln et al., 2007; Mierke et al., 2017; Kunschmann et al., 2017, 2019). More precisely, the measurement procedure was as follows: a single cell was trapped for 1 s between two each-other facing divergent laser beams at a low laser power of 100 mW, deformed by increasing laser powers up to 800 mW or 1200 mW for 2 s and finally the laser powers were reduced to 100 mW to record the relaxation behavior for 2 s (Guck et al., 2001). All analyzed cells showed a creep behavior as response to the deformation of the cell in parallel to the laser beam axes. The measurement procedure was the same for all measurements and the temperature was kept constant to 23°C.

For cell deformation measurements, the inhibition of PAK was performed with two pharmacological drugs, such as FRAX597 and IPA3 (Sigma Aldrich, Germany). To determine the optimal inhibitor concentration, various concentrations were tested and the most effective concentration was determined in which cells were still viable. Hence, cells were treated 2 h before measurement start with 1.2 μM FRAX597 or 12 μM IPA3 and the optical cell stretcher measurements were performed with each of the two drugs and with the solvent of control vehicle. Measurements were repeated at least three times for each condition. The optical cell stretcher experiments have been performed three times independently and cell numbers were between 1403 and 2122 for each condition. In specific detail, for the FRAX597 inhibitor experiments: Rac1^fl/fl^ control cells at 800 mW (*n* = 2100 cells) and at 1200 mW (*n* = 2100 cells); Rac1^fl/fl^ cells treated with FRAX597 at 800 mW (*n* = 2090 cells) and at 1200 mW (*n* = 2110 cells); Rac1^–/–^ control cells at 800 mW (*n* = 1870 cells) and at 1200 mW (*n* = 1683 cells); Rac1^–/–^ cells treated with FRAX597 at 800 mW (*n* = 2078 cells) and at 1200 mW (*n* = 2122 cells). For the IPA3 inhibitor experiments: Rac1^fl/fl^ control cells at 800 mW (*n* = 1422 cells) and at 1200 mW (*n* = 1378 cells); Rac1^fl/fl^ cells treated with IPA3 at 800 mW (*n* = 1403 cells) and at 1200 mW (*n* = 1397 cells); Rac1^–/–^ control cells at 800 mW (*n* = 2170 cells) and at 1200 mW (*n* = 2073 cells); Rac1^–/–^ cells treated with IPA3 at 800 mW (*n* = 2117 cells) and at 1200 mW (*n* = 2083 cells).

### Data Analysis of Cellular Deformation

An automated subpixel edge detection algorithm implemented in MathLab (Math Works, Guck et al., 2001, 2005) was employed to determine relative cell deformations during optical cell stretcher measurements. In detail, small angle rotations of trapped cells were corrected by feature tracking and irregularly shaped cells were excluded. In contrast, large angel rotation during cell deformation results in wrong relative deformation results and were excluded. The remaining cells were analyzed with respect to their creep behavior J(t) = ε(t)/σ_0_. (t) = [d(t)-d(0)]/d(0) represents the relative cell deformation (strain) along the laser beam axes and σ_0_ is the optical induced stress that depends linearly on the stretch laser power (Guck et al., 2000, 2005). The obtained maximum deformation values were presented as median values and the bootstrapping method was employed to estimate a 95.46% confidence interval (2^∗^SD). A large number of cells was measured in each experiment in order to obtain reliable and reproducible results representing of the entire cell population.

### Statistical Analysis

**FIGURE 1:**
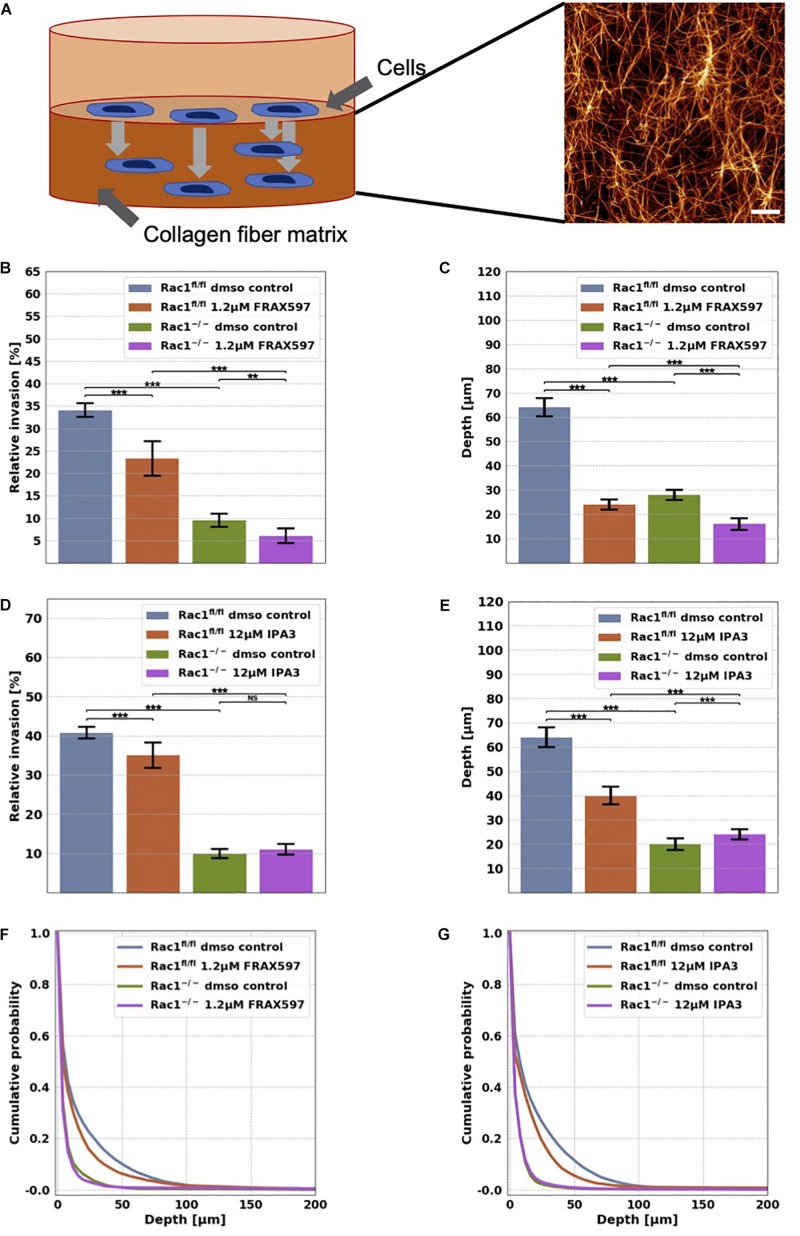
Migration and invasion of Rac1^fl/fl^ and Rac1^–/–^ cells in confined 3D extracellular matrices. **(A)** Schematic illustration of a 3D extracellular matrix assay and a representative laser scanning confocal image of a 1.5 g/l 3D collagen fiber matrix stained with TAMRA. Scale bar is 20 μm. **(B)** Average percentage of invasive cells and their invasion depth **(C)** of Rac1^fl/fl^ and Rac1^–/–^ cells treated 72 h with 1.2 μM FRAX597. The percentage of invasive cells and their invasion depth of FRAX597 treated cells was decreased compared to control treated cells. **(D)** Average percentage of invasive cells and their invasion depth **(E)** of Rac1^fl/fl^ and Rac1^–/–^ cells treated 72 h with 12 μM IPA3. The percentage of invasive cells and invasion depth of IPA3 treated Rac1^fl/fl^ cells was decreased compared to control treated cells, whereas the relative invasion of Rac1^–/–^ cells was not altered by IPA3. Cumulative probability over invasion depth of Rac1^fl/fl^ **(F)** and Rac1^–/–^ cells **(G)** stimulated with 1.2 μM FRAX597 and 12 μM IPA3, respectively. A *p*-value below 0.05 is considered as statistically significant, ***p < 0.001, **p < 0.01 and NS, not significant.

**FIGURE 2:**
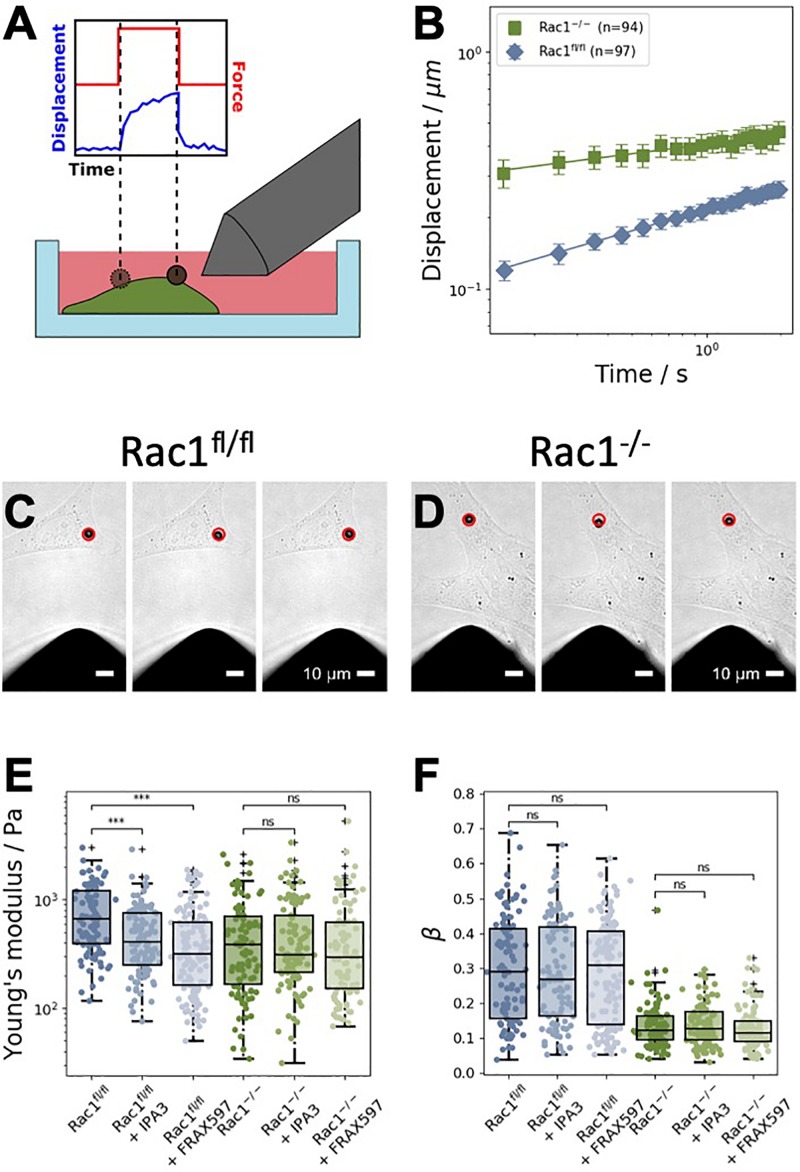
Magnetic tweezer measurements of Rac1^fl/fl^ and Rac1^–/–^ cells. **(A)** Schematic representation of the measurements. Fibronectin-coated beads were coupled to the cell’s surface. A constant force of one nanonewton was applied for 2 s to displace the bead. **(B)** Averaged displacement curves of Rac1^fl/fl^ and Rac1^–/–^ cells in a double-logarithmic scale. The displacement curves closely followed a weak power law. **(C)** Representative brightfield images of a 4.5 μm superparamagnetic bead coupled to a Rac1^fl/fl^ cell. The red circle in all images marks the initial position of the bead. Scalebars are 10 μm. Left: Bead position just before the force is turned on. Middle: Bead position after 2 s of force application. Right: Bead position after 2 s of relaxation after the force is turned off. **(D)** Representative brightfield images of a Rac1^–/–^ cell were taken at the same time stamps as for the Rac1^fl/fl^ cell. The maximal bead displacement in Rac1^–/–^ cells is generally stronger than in Rac1^fl/fl^ cells. **(E)** After treatment with different PAK inhibitors, such as IPA3 and FRAX597, the Young’s modulus of Rac1^fl/fl^ cells was decreased compared to buffer treated control cells. Treatment with the inhibitors had no effect on the Young’s modulus of Rac1^–/–^ cells. **(F)** For both Rac1^fl/fl^ and Rac1^–/–^ cells, the viscoelastic state β (i.e., the power law exponent β) was unaffected by treatment with IPA3 and FRAX597. ***p < 0.001 and ns, not significant.

All experimental data were presented as median values unless otherwise stated. The statistical analyses were performed by using the Kruskal–Wallis test, since we have unequal variances. It is included as Supplementary Material for the Figures 1, 2, 4, and 5 (Supplementary Material). Additionally, we performed Bonferroni corrections on the individual hypotheses to further increase the statistical power of our analysis. In general, *p*-values of 0.05 were considered as statistically significant. It was marked with a single star, *p*-values of 0.01 were marked with two stars and *p*-values of 0.001 were highlighted with three stars.

**FIGURE 3:**
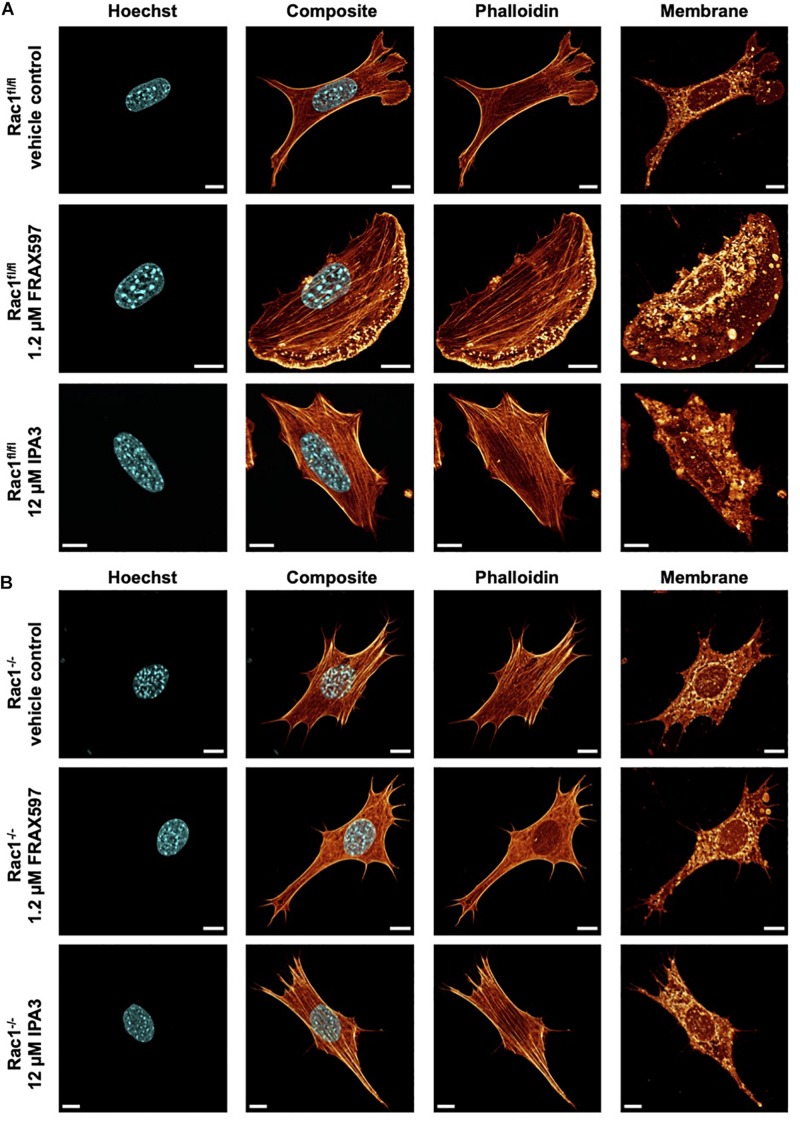
Inhibition of group I PAKs and PAK1 by FRAX597 and IPA3 alters the morphology and the actin cytoskeleton of Rac1^fl/fl^ and Rac1^–/–^ cells using a confocal laser scanning microscopy. Cells were cultured on planar substrates coated with 10 μg/ml laminin and treated for 2 h with 1.2 μM FRAX597 or 12 μM IPA3. After fixation, the cells were stained with Alexa Fluor 546 phalloidin, Hoechst and DID. **(A)** Nuclear shape, composite of nuclear shape and action cytoskeleton, actin cytoskeleton and membrane shape of a representative Rac1^fl/fl^ cell in absence (top row) and presence of FRAX597 (intermediate row) or IPA3 (bottom row) is presented. **(B)** Nuclear shape, composite of nuclear shape and action cytoskeleton, actin cytoskeleton and membrane shape of a representative Rac1^–/–^ cell in absence (top row) and presence of FRAX597 (intermediate row) or IPA3 (bottom row) is presented. All scale bars are 10 μm.

**FIGURE 4:**
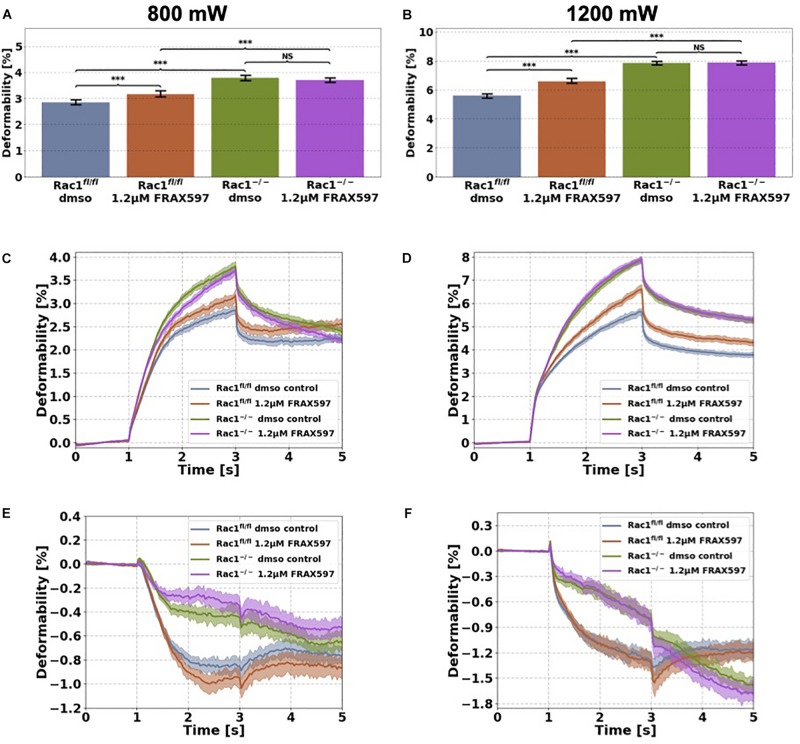
Cellular stiffness (inverse deformability) of non-adhesive Rac1^fl/fl^ and Rac1^–/–^ cells stimulated with 1.2 μM FRAX597 using an optical cell stretcher. Cells are trapped for 1 s at 100 mW laser powers and then stretched for 2 s by increasing laser powers up to 800 mW **(A,C,E)** or 1200 mW **(B,D,F)** along laser beam axes. After stretching process, laser powers were reduced to 100 mW and viscoelastic relaxation was observed for 2 s. Maximal deformation of Rac1^fl/fl^ cells is pronouncedly increased (stiffness decreased) by 1.2 μM FRAX597 for 800 mW **(A)** and 1200 mW **(B)** laser powers. Maximal deformation of Rac1^–/–^ cells was unaffected by 1.2 μM FRAX597 for both laser powers. The data are presented as median with SD. Cellular deformation along the long axis (parallel to laser beam axes) of Rac1^fl/fl^ and Rac1^–/–^ cells treated with 1.2 μM FRAX597 for 2 h at laser powers of 800 mW **(C)** and 1200 mW **(D)**. Mechanical behavior along the short axis (perpendicular to laser beam axes) of Rac1^fl/fl^ and Rac1^–/–^ cells treated for 2 h with 1.2 μM FRAX597 at laser powers of 800 mW **(E)** and 1200 mW **(F)**. Data are presented as median values with confidence interval (2*SD) of 95.46%. A *p*-value of 0.05 is considered as statistically significant, ***p < 0.001 and NS, not significant.

**FIGURE 5:**
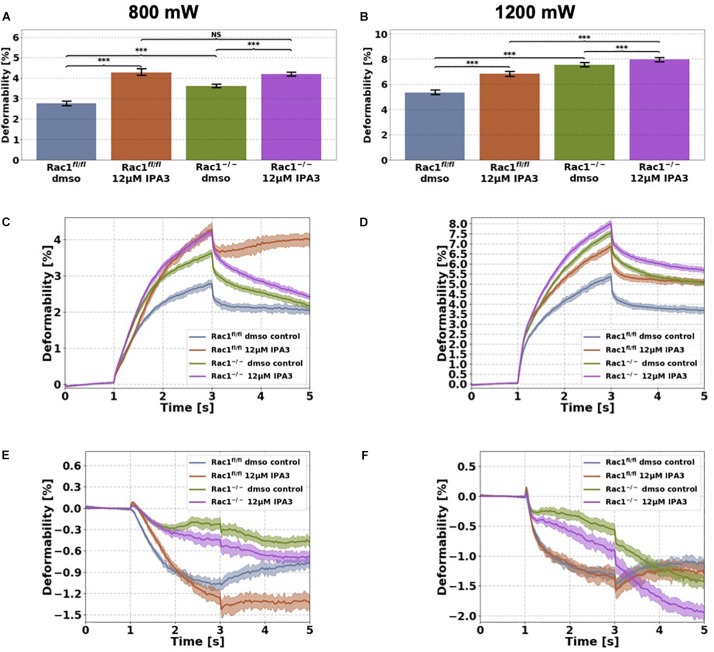
Cellular stiffness (inverse deformability) of non-adhesive Rac1^fl/fl^ and Rac1^–/–^cells stimulated with 12 μM IPA3 using an optical cell stretcher. Cells are trapped at 100 mW laser powers for 1 s and then stretched for 2 s by increasing laser powers up to 800 mW **(A,C,E)** or 1200 mW **(B,D,F)** along laser beams axes. After stretching process, laser powers were reduced to 100 mW and viscoelastic relaxation was observed for 2 s. **(A,B)** Maximal deformation of Rac1^fl/fl^ is pronouncedly increased (stiffness decreased) by 12 μM IPA3 for both laser powers. Rac1^–/–^ cells showed an increased maximal deformation (stiffness decreased) for 800 mW **(A)** and only a slightly increased deformation for 1200 mW **(B)**. The data are presented as median with SD. Cellular deformability along the long axis of Rac1^fl/fl^ and Rac1^–/–^ cells incubated with 12 μM IPA3 for 2 h at laser powers of 800 mW **(C)** and 1200 mW **(D)** were presented. Mechanical behavior along the short axis of Rac1^fl/fl^ and Rac1^–/–^ cells incubated for 2 h with 12 μM IPA3 at laser powers of 800 mW **(E)** and 1200 mW **(F)**. Data are presented as median values with confidence interval (2*SD) of 95.46%. A *p*-value of 0.05 is considered as statistically significant, ***p < 0.001 and NS, not significant.

## Results

### Inhibition of PAK Reduces the Invasiveness of Rac1^fl/fl^ Into 3D Extracellular Matrices, Whereas the Invasiveness of Rac1^–/–^ Cells Is Slightly Altered

In order to investigate the effect of group I PAKs on cell migration into 3D microenvironments, we performed invasion assays into 3D extracellular matrices with Rac1^fl/fl^ and Rac1^–/–^ cells that were treated either with 1.2 μM FRAX697 or 12 μM IPA3. The 3D matrix scaffold consists of a mixture of 1/3 rat tail collagen type I and 2/3 bovine skin collagen type I that polymerizes to a proper network of collagen fibers and bundles (Figure 1A). Rac1^fl/fl^ and Rac1^–/–^ cells were seeded on top of the 3D extracellular matrices with a concentration of 1.5 g/l and a thickness of 500 μm. They were incubed for 12 h to adhere on these matrices. To reveal whether group I PAKs regulate the invasion behavior of Rac1^fl/fl^ and Rac1^–/–^ cells, we treated these cell types with 1.2 μM FRAX697 or 12 μM IPA3. Control cells of each cell type were treated with solvent of control vehicle (DMSO). Cells invaded for 72 h and the percentage of invaded cells and their invasion depth were determined. The percentage of invasive cells and invasion depths of Rac1^fl/fl^ cells were reduced by both inhibitory drugs 1.2 μM FRAX597 and 12 μM IPA3 (Figures 1B–G). Invasiveness and invasion depth of Rac1^–/–^ cells was also decreased by 1.2 μM FRAX597 (Figures 1B,C), whereas a stimulation with 12 μM IPA3 had no significant effect on their percentage of invasive cells (Figures 1D,F). Whereas invasion depth of Rac1^–/–^ cells was even slightly increased by 12 μM IPA3 (Figures 1E,G). These results indicate that PAK1 inhibition by FRAX597 and IPA3 impaired significantly the invasiveness of Rac1^fl/fl^ cells in 3D extracellular matrices, whereas Rac1^–/–^ cells were only slightly impaired (FRAX597) or even slightly increased (IPA3) in invasiveness by the inhibition of group I PAKs indicating that they may use a different migration mode.

### Effect of Group I PAKs Inhibition on the Stiffness of Adhesive Cells

In order to examine the effect of group I PAKs inhibition in the presence or absence of Rac1, we employed the magnetic tweezer technique to determine cell stiffness and fluidity. Therefore, we used Rac1^fl/fl^ and Rac1^–/–^ cells to analyze the cell mechanical properties in the presence and absence of Rac1 in adhesive cells. In detail, we bound fibronectin-coated beads to the adhesive cell types and run the following measurement protocol: recording of the bead displacement for 1 s (background noise), for 2 s, when the force is switched on (by switching on the current in the coil of the tweezer needle) and for another 2 s, when the force is switched off (relaxation phase, Figure 2A). The displacement of the beads bound to Rac1^–/–^ cells was significantly larger than that of beads bound to Rac1^fl/fl^ cells (Figure 2B). Hence, the adhesive Rac1^–/–^ cells were less stiff (softer and more deformable) compared to adhesive Rac1^fl/fl^ cells. This result is in line with deformability measurements of the two cell types using optical cell stretching, where the non-adhesive Rac1^–/–^ cells were less stiff compared to non-adhesive Rac1^fl/fl^ cells (Kunschmann et al., 2019). The images in Figures 2C,D show the bead displacement of a representative cell for both cell types during the three measurement phases, such as force off (background), force on (force application) and force off (relaxation) phases. Both PAK inhibitors reduced the stiffness of Rac1^fl/fl^ cells significantly, whereas the stiffness of Rac1^–/–^ cells was not altered (Figure 2E). More precisely, the effect of FRAX597 was more pronounced compared to IPA3 in Rac1^fl/fl^ cells. However, the cell fluidity (power law exponent β) of the two cell types was not affected by treatment of the two different PAK inhibitors (Figure 2F).

### PAK Inhibition Alters Morphology and Actin Cytoskeleton of Rac1^fl/fl^ and Rac1^–/–^ Cells

For a correlation of decreased invasiveness and increased cellular deformation under inhibition of group I PAKs with the cell shape, we investigated the effect of FRAX597 and IPA3 on the morphology and actin cytoskeleton of Rac1^fl/fl^ and Rac1^–/–^ cells. Therefore, we cultured both cell types on planar substrates coated with 10 μg/ml laminin and treated either with 1.2μM FRAX597 or 12 μM IPA3 for 2 h. After fixation, cells were stained with Alexa Fluor 546 phalloidin, Hoechst and DID. A confocal laser scanning microscope was used to analyze the actin cytoskeleton and the morphology of the cells by recording *z*-stacks with a *z*-distance between neighboring images of approximately 130–200 nm. We observed that IPA3 impaired lamellipodia formation in Rac1^fl/fl^ cells to an extent similar to Rac1^–/–^ cells in all fields of view (Figure 3A). Moreover, Rac1^fl/fl^ cells displayed a less branched actin network and exhibited increased aligned actin fibers (Figure 3A bottom row). Lamellipodia formation was still present and even slightly enhanced under FRAX597 treatment and the actin network appeared to be scattered consisting of coarser actin bundles. Actin fibers seem to end in a more condensed form in lamellipodia in all fields of view (Figure 3A intermediate row). However, we have currently no explanation for this. In contrast, Rac1^–/–^ cells displayed no obvious differences under IPA3 treatment inhibiting most efficiently PAK1 (Figure 3B bottom row). Similarly, inhibition of group I PAKs by FRAX597 did not have an effect on cell morphology, nuclear shape or the actin cytoskeleton (Figure 3B intermediate row).

### Inhibition of Group I PAKs by FRAX597 Decreases Cellular Stiffness of Non-adhesive Rac1^fl/fl^ Cells

We used the optical cell stretcher to determine the effect of group I PAKs, they are inhibited by FRAX597 on cellular stiffness (inverse deformability) of Rac1^fl/fl^ and Rac1^–/–^ cells. The optical cell stretcher is a dual beam laser trap that can deform single non-adhesive cells by laser induced optical forces. Cells are trapped and deformed between two opposing divergent laser beams. Cells are transported by a microfluidic pump system to the region of interest and trapped at low laser powers of 100 mW for 1 s (trap phase). Cells are then deformed by increasing the laser powers in a step like manner from 100 mW up to 800 mW or 1200 mW for 2 s (stretch phase). In the last step, laser powers were reduced to 100 mW and cell relaxation was observed for another 2 s (relaxation phase). All experiments for each condition were performed at least three times.

Cells were stimulated 2 h before measurement with 1.2 μM FRAX597. The deformation of major (long) cell axis parallel to laser beam axis displayed a time-dependent creep behavior J(t) (Figure 4). The median creep deformations (maximal deformations) at time point 3 s at the end of each stretch phase J(*t* = 3 s) were determined to compare the mechanical deformability (representing maximal deformation) of the cells under PAK inhibition (Figures 4A,B). Rac1^fl/fl^ cells exhibited increased cellular deformability (decreased stiffness) of their long axis under group I PAKs inhibition for laser powers of 800 mW compared to control treated cells (Figures 4A,C). In contrast, cellular deformability of Rac1^–/–^ cells was not affected by group I PAKs inhibitor FRAX597 for 800 mW laser powers (Figures 4A,C). Similar results were determined for laser powers of 1200 mW, where FRAX597 treatment decreased the stiffness of Rac1^fl/fl^ cells significantly, whereas the stiffness of Rac1^–/–^ cells was not altered (Figures 4B,D). The relaxation behavior of Rac1^fl/fl^ cells was slightly modulated for laser powers of 800 and 1200 mW after FRAX597 treatment (Figure 4C).

The shrinkage behavior of minor axis (perpendicular to laser beam axes) of both cell types was not significantly altered by FRAX597 treatment (Figures 4E,F). These results demonstrate that PAK inhibition by FRAX597 regulates cellular deformability (invers stiffness) and increases cellular deformation (decreases cell stiffness) of Rac1^fl/fl^ cells, whereas Rac1^–/–^ cells are not affected. Subsequently, our results indicate that impairing the kinase domain of group I PAKs has only an impact on cell stiffness, when the cells express Rac1.

### Inhibition of PAK1 by IPA3 Decreases Cellular Stiffness of Non-adhesive Rac1^fl/fl^ and Rac1^–/–^ Cells

Since interference of the kinase domain of group I PAKs by FRAX597 decreases cell stiffness (increases cell deformability) of Rac1^fl/fl^ cells, we examined the effect of blocking the structural function of PAK1 by IPA3 of the two cell types in their non-adhesive state using the optical cell stretcher. As expected, we found that the cellular deformability of Rac1^fl/fl^ cells is significantly increased (and inverse decreased stiffness) after IPA3 treatment for both used laser powers (Figures 5A–D). In addition, cellular deformability of Rac1^–/–^ cells was also slightly increased by IPA3 treatment for 800 mW (Figure 5C), whereas the effect was less pronounced at laser powers of 1200 mW (Figure 5D). In detail, the maximal deformation was significantly increased for both cell types and laser powers (Figures 5A,B). The relaxation behavior of Rac1^fl/fl^ cells along the long axis was strongly affected by IPA3 treatment for laser powers of 800 mW (Figure 5C). However, a similar behavior was not detected for laser powers of 1200 mW. The deformation behavior of Rac1^fl/fl^ cells and Rac1^–/–^ cells along the short (minor) axis was slightly affected by IPA3 treatment for low laser powers of 800 mW (Figure 5E). Additionally, Rac1^fl/fl^ cells showed a similar relaxation behavior as Rac1^–/–^ cells along the perpendicular axis for low laser powers (Figure 5E). This effect was not seen for high laser powers of 1200 mW (Figure 5F). Finally, we found that an inhibition of the PAK1 by IPA3 increases cellular deformability of Rac1^fl/fl^ cells pronouncedly and also slightly in Rac1^–/–^ cells indicating that the structural function of PAK1 group members can still be seen in Rac1 knock-out cells.

## Discussion

The migration and invasion through confined 3D microenvironments such as connective tissue seem to rely, apart from biochemical factors, on the mechanical properties of cells. In many diseases, such as wound healing after tissue injury or cancer metastasis, cell migration and invasion are essential and thereby the cells face confined environments, in which the cells need to exhibit a specific mechanical phenotype to migrate through them. Therefore, it is important to investigate the role of the mechanical properties of cells on their functions, such as cell motility. More precisely, it needs to be determined what role group I PAKs and especially PAK1 play in providing cell mechanics. A mechanical property, which is explored in this study, is the cell Young’s modulus or stiffness (inverse deformability) that is considered to be crucial for cell migration and invasion in confinements. Using a previously established 3D collagen fiber matrix invasion assay, we determined the effect of PAK on cell migration and invasion in the presence or absence of Rac1. In addition, the mechanical probing techniques that can be either employed to adhesive cells, such as magnetic tweezers, or to non-adhesive cells, such as optical cell stretching, were utilized to reveal whether the group I PAKs (FRAX597) and PAK1 inhibition (IPA3) affect the cell mechanical properties in the presence or absence of Rac1.

The inhibition of PAK with both FRAX597 (group I PAKs) and IPA3 (PAK1) inhibitors causes a pronounced decrease in the percentage of invasive cells in Rac1^fl/fl^ cells and a pronounced reduction in the invasion depths of these invasive cells. These results are in line with the 3D invasion results obtained by knock-out of Rac1 in fibroblasts (Kunschmann et al., 2019) and also with 2D migration assays (Steffen et al., 2013). However, there exists differences in FRAX597 and IPA3 inhibition of group I PAKs and PAK1, respectively, since the inhibition of IPA3 affects the structural function of PAK1 and thereby has an effect on the percentage of invasive cells and the invasion depths of Rac1^–/–^ cells. FRAX597 solely impairs the kinase domain of PAK1-3 interfering with the interaction of Rac1 or Cdc42 with PAK1-3. It is known that group I PAKs and PAK1 fulfill several major roles, which can be either kinase-dependent and kinase-independent (structure-dependent function). Since FRAX597 binds non-covalently to the ATP-binding site of group I PAKs and thereby impairs their kinase activity (Licciulli et al., 2013), the effect caused by FRAX597 reveals its kinase activity-dependent function. In contrast, IPA3 represents an allosteric inhibitor that specifically interacts with the inactive PAK1 form and thus probably also blocks non-kinase functions of these proteins. Moreover, PAK1 can facilitate the connection to microtubules (LaFlamme et al., 2018; Hohmann and Dehghani, 2019), which may additionally alter the Young’s modulus or cell stiffness independent of Rac1 in both cell types. The addition of IPA3 to Rac1^–/–^ cells even increased the percentage of invasive cells and their invasion depths indicating that these cells can utilize a different migration mode, which may possibly rely on cell squeezing through the network. However, further investigations are required to reveal a mechanism.

When probing cell mechanics of the two cell types, it can be suggested that cell adhesion plays a major role on cytoskeletal dynamics and subsequently cell mechanics (Stossel et al., 1985; DiMilla et al., 1991; Wakatsuki et al., 2003; Fischer et al., 2017). Hence, we determined for the first time the cell mechanics of the two cell types using both magnetic tweezers (adhesive cells) and optical cell stretching (non-adhesive cells) in a combined study. In fact, we found that the adhesive state of the cell during the cell mechanical analysis is only minor, since the stiffness of Rac1^fl/fl^ cells was in both cell mechanical techniques significantly increased compared to Rac1^–/–^ cells. Moreover, even the inhibition of group I PAKs and PAK1 in these two cell types revealed similar results, which again indicate that the adhesive state of the cells has only a minor influence on cell mechanics. However, when using the IPA3 inhibitor of PAK1, which impairs its structural function, there is a difference in the behavior of adhesive and non-adhesive Rac1^–/–^ cells indicating that the structural function of PAK1 may be cell adhesion state dependent. Another explanation may be the fact that the structural interaction of PAK1 with microtubules is blocked leading to decreased stiffness of Rac1 knock-out cells in the presence of IPA3 in their non-adhesive state.

There are differences in the effect of the two PAK inhibitors FRAX597 and IPA3 between the two cell types, since FRAX597 that impairs the kinase domain (by competing with ATP binding) alters only the cell mechanics of Rac1^fl/fl^ cells, whereas that of Rac1^–/–^ cells are not affected.

Overall, this study demonstrates the importance of examining the effect of group I PAKs and PAK1 inhibition on cell mechanics in response to presence or absence of Rac1. It is becoming increasingly evident that these cell mechanics can provide cell migration and invasion of normal healthy cells and diseased pathological cells. Understanding the impact of group I PAKs and PAK1 and Rac1 to cell mechanics and invasion can lead to identification of mechanotransduction processes for future treatments to modify cell migration under certain circumstances, such as cancer metastasis or wound healing processes after tissue injury.

## Data Availability Statement

The raw data supporting the conclusion of this article will be made available by the authors, without undue reservation, to any qualified researcher.

## Author Contributions

CM had the idea for the manuscript, interpreted the data, prepared figures, and wrote the whole manuscript. SP analyzed the data, prepared figures, and helped in writing results. CA performed the magnetic tweezer measurements, analyzed the data, and prepared a figure. TF analyzed and interpreted the data. TK performed all other experiments, analyzed the data, and prepared figures.

## Conflict of Interest

The authors declare that the research was conducted in the absence of any commercial or financial relationships that could be construed as a potential conflict of interest.
