# Supplementary material for: Effect of PAK Inhibition on Cell Mechanics Depends on Rac1
Source: Front Cell Dev Biol. 2020 Jan 28;8:13. doi: 10.3389/fcell.2020.00013 (PMC6997127; doi:10.3389/fcell.2020.00013)
Supplement: Supplementary file 1 [file Table_1.docx]

Supplementary Material

**Supplementary Table 1:** Statistical analysis of the invasion data of figure 1 using the Kruskal-Wallis test.

| **Cell line** | **value** | **p-value** | **h-statistic** | **significance** |
| --- | --- | --- | --- | --- |
| **Rac1^fl/fl^ vs Rac1^-/-^**  **FRAX597** | invasiveness | 1.81 E-114 | 267.67022 | *** |
| **Rac1^fl/fl^ vs Rac1^-/-^**  **FRAX597** | depth | 3.99 E-121 | 320.970081 | *** |
| **Rac1^fl/fl^ vs Rac1^-/-^**  **IP3** | invasiveness | 1.92 E-195 | 485.23022 | *** |
| **Rac1^fl/fl^ vs Rac1^-/-^**  **IP3** | depth | 1.40 E-228 | 649.09700 | *** |

**Supplementary Table 2:** Statistical analysis of the magnetic tweezer data of figure 2 using the Kruskal-Wallis test.

| **Cell line** | **value** | **p-value** | **h-statistics** | **significance** |
| --- | --- | --- | --- | --- |
| **Rac1^fl/fl^** | Stiffness | 1,80E-08 | 35,66357 | *** |
| **Rac1^fl/fl^** | beta | 0,84877 | 0,32793 | ns |
| **Rac1^-/-^** | Stiffness | 0,37486 | 1,96239 | ns |
| **Rac1^-/-^** | beta | 0,22295 | 3,00159 | ns |
| **Rac1^fl/fl^ + Rac1^-/-^** | Stiffness | 8,18095 | 49,22288 | *** |
| **Rac1^fl/fl^ + Rac1^-/-^** | beta | 1,67E-37 | 182,36196 | *** |

**Supplementary Table 3:** Statistical analysis of the optical stretcher data of figures 4 and 5 using the Kruskal-Wallis test.

|  | **drug** | **laser power** | **p-value** | **h-statistics** | **significance** |
| --- | --- | --- | --- | --- | --- |
| **0** | 1.2 µM FRAX597 | 800 | 4,48E-73 | 338,5586 | *** |
| **1** | 1.2 µM FRAX597 | 1200 | 2,5E-156 | 722,7149 | *** |
| **2** | 12 µM IPA3 | 800 | 4,16E-98 | 454,1277 | *** |
| **3** | 12 µM IPA3 | 1200 | 1,4E-163 | 756,1667 | *** |
